# A new fetal RHD genotyping test: Costs and benefits of mass testing to target antenatal anti-D prophylaxis in England and Wales

**DOI:** 10.1186/1471-2393-11-5

**Published:** 2011-01-18

**Authors:** Ala Szczepura, Leeza Osipenko, Karoline Freeman

**Affiliations:** 1 Warwick Medical School, University of Warwick, Coventry, UK

## Abstract

**Background:**

Postnatal and antenatal anti-D prophylaxis have dramatically reduced maternal sensitisations and cases of rhesus disease in babies born to women with RhD negative blood group. Recent scientific advances mean that non-invasive prenatal diagnosis (NIPD), based on the presence of cell-free fetal DNA in maternal plasma, could be used to target prophylaxis on "at risk" pregnancies where the fetus is RhD positive. This paper provides the first assessment of cost-effectiveness of NIPD-targeted prophylaxis compared to current policies.

**Methods:**

We conducted an economic analysis of NIPD implementation in England and Wales. Two scenarios were considered. Scenario 1 assumed that NIPD will be only used to target antenatal prophylaxis with serology tests continuing to direct post-delivery prophylaxis. In Scenario 2, NIPD would also displace postnatal serology testing if an RhD negative fetus was identified. Costs were estimated from the provider's perspective for both scenarios together with a threshold royalty fee per test. Incremental costs were compared with clinical implications.

**Results:**

The basic cost of an NIPD in-house test is £16.25 per sample (excluding royalty fee). The two-dose antenatal prophylaxis policy recommended by NICE is estimated to cost the NHS £3.37 million each year. The estimated threshold royalty fee is £2.18 and £8.83 for Scenarios 1 and 2 respectively. At a £2.00 royalty fee, mass NIPD testing would produce no saving for Scenario 1 and £507,154 per annum for Scenario 2. Incremental cost-effectiveness analysis indicates that, at a test sensitivity of 99.7% and this royalty fee, NIPD testing in Scenario 2 will generate one additional sensitisation for every £9,190 saved. If a single-dose prophylaxis policy were implemented nationally, as recently recommended by NICE, Scenario 2 savings would fall.

**Conclusions:**

Currently, NIPD testing to target anti-D prophylaxis is unlikely to be sufficiently cost-effective to warrant its large scale introduction in England and Wales. Only minor savings are calculated and, balanced against this, the predicted increase in maternal sensitisations may be unacceptably high. Reliability of NIPD assays still needs to be demonstrated rigorously in different ethnic minority populations. First trimester testing is unlikely to alter this picture significantly although other emerging technologies may.

## Background

In white Caucasian populations about 10% of all pregnancies involve a mother with rhesus (Rh) D negative blood group and an RhD positive fetus, potentially placing the mother at risk of sensitisation and future babies at risk of haemolytic disease of the fetus and newborn. Anti-D prophylaxis (anti-D IgG) can be given to prevent a woman producing antibodies against fetal RhD-positive blood cells and becoming sensitised. Prophylaxis following delivery was introduced in the 1960s, with a blood cord serology test used to identify the baby's RhD status. This dramatically reduced maternal sensitisations and cases of rhesus disease in babies [1]. In the mid-1990s, routine antenatal anti-D prophylaxis (RAADP) was first used. This was reported to further reduce sensitisation rates (from 1.2% for the earlier policy to 0.28%) [2], with RAADP stated to be 98.4-99% effective [3]. In 2002, the National Institute for Health and Clinical Excellence (NICE) published guidelines for the UK, recommending two doses (500iu each) of anti-D IgG at weeks 28 and 34 of gestation as effective and cost-effective [1]. Figures indicate that 90% of hospitals in England and Wales comply with these guidelines, with 90% of the target population reported to receive the first dose of anti-D IgG and up to 87% the second dose [4,5]. In 2008, updated NICE guidance stated that a single dose of anti-D (1500iu) between weeks 28 and 30 would also be cost-effective [6]. However, with both RAADP policies, the 40% of RhD negative women whose fetus is also RhD negative will receive antenatal prophylaxis unnecessarily [1].

Non-invasive prenatal diagnosis (NIPD) of fetal *RHD *blood group is based on the presence of cell-free fetal DNA in maternal plasma [7-10]. Fetal *RHD *genotyping of this material has the potential to enable antenatal prophylaxis targeted at the 60% of pregnancies with an RhD positive fetus, thereby saving anti-D costs. NIPD test accuracy figures in the range 94.8% - 100% have been reported [8,9,11-14], although studies exhibit certain shortcomings [15]. By 2007, many European countries had introduced NIPD testing for the small number of sensitised women in order to identify high risk pregnancies (fetus RhD positive) [10,14,16]. Approximately 250-300 sensitised women in England and Wales now undergo RhD NIPD tests annually. In such cases the NIPD test offers the clinical advantage of avoiding an invasive procedure such as amniocentesis with its associated risk of fetal loss [17], as well as possible cost savings.

Several authors have recently recommended a wider roll-out of NIPD testing to the remaining, non-sensitised pregnancies [10,14,16], with suggestions that this will be cost saving [16,18]. Such an approach would extend testing to a significantly larger population. Approximately 16% of white women are RhD negative; RhD negativity in other ethnic groups is lower; South Asians (5.5% - 10.9%), West Africans (5%), Chinese (<1%) [19,20]. Extension of NIPD testing to this larger population (approximately 93,000 pregnancies annually in England and Wales [21]) is not feasible with the low throughput and labour intensive processes used for testing samples from sensitised women [22]. Mass testing will require an accurate automated laboratory procedure, similar to that recently reported which has a test sensitivity of 99.7% [16]. NIPD testing is not able to identify the degree to which a woman is prone to sensitisation because this is governed by several factors in addition to fetal *RHD *status [23].

To date the costs and implications of large scale NIPD testing to target antenatal prophylaxis have not been determined. The aim of the present study was to estimate the cost savings (if any) in England and Wales for two implementation strategies compared to the current two-dose RAADP programme (and a possible future single-dose policy). The research was undertaken as part of the SAFE Network of Excellence funded by the European Commission [24].

## Methods

### Design of the study

Costs were estimated for the following strategies for implementing NIPD:

• *Scenario 1: *Assumed that all RhD negative women will routinely receive an NIPD test and that antenatal prophylaxis will be *withheld *if an RhD negative fetus is identified; post-delivery testing and postnatal prophylaxis assumed to be *unaffected*.

• *Scenario 2: *Assumed that, in addition to Scenario 1, post-delivery blood cord serology and associated Kleihauer test (to measure the amount of fetal haemoglobin transferred to the mother's bloodstream to determine anti-D IgG dose required) will *be withheld *if NIPD has identified an RhD negative fetus.

The cost analysis was undertaken from the perspective of the National Health Service (NHS healthcare provider) in England and Wales. It was assumed that NIPD testing could be fitted into current antenatal care without increasing the number of prenatal visits, so costs such as patient time, travel etc. were not expected to differ significantly. A primary costing approach and financial modelling were used to estimate the incremental cost of moving from the status quo (RAADP) to one of the two scenarios. The consequences of implementing each scenario were also estimated, based on a range of parameters. All costs are expressed in 2009 prices.

### Test costs

A unit test cost (base case) was determined for NIPD. Laboratory resource use data were collected in the International Blood Group Reference Laboratory, Bristol, UK [16]. In-house laboratory processing costs were based on a protocol developed and standardised in several European laboratories [25]. Equipment costs, including plasma preparation, DNA extraction (MDX robot from Qiagen or similar) and DNA sequencing (7900 SD from Applied Biosystems or similar) were discounted over five years and converted to an annual equivalent cost; associated annual maintenance contract costs were added. A maximum annual throughput of 44,000 samples per set of equipment was assumed. At current RAADP uptake levels in England and Wales, two sets of equipment would be required; if all RhD negative pregnancies were to be tested, a third set of equipment would be needed. Consumable list prices for primers, probes (Exon 5 and 7), controls, reagents and other items were discounted by 20% to allow for bulk purchase. Direct staff time (excluding non-contact time) included employer on-costs (NI and Superannuation) at 22% [26]. Interpretation of results was assumed to be carried out by an automated Hematos system [27]. Laboratory overheads were assumed to be similar to other tests at the study site. Allowance was made for inconclusive NIPD results requiring re-test rates between 1.4% and 3% [10,16,28]. Re-test costs assumed no further phlebotomy cost with some original blood sample preserved for re-tests. Sample collection costs included consumables and staff time [29]. The cost of sample transport was based on local transport system prices. Postnatal serology test, Kleihauer test and associated phlebotomy costs were based on local prices (West Midlands).

An in-house testing service might also incur a royalty payment (fetal DNA presence in maternal blood: United States Patent 6258540). This has not yet been negotiated in any country. An alternative to an in-house test would be use of a commercial kit (Institut de Biotechnologies Jacques Boy, France) which has recently been CE marked for use in Europe [30]. The price of the kit includes a royalty fee, controls, primers, probes (Exons 7, 10 and IRV2) but excludes other materials (MDX kit, LG tips, SML tips, Eton, AW1, buffer, detergent). A unit test cost was similarly calculated for NIPD testing using this commercial kit.

### Cost of Status Quo Antenatal Anti-D Prophylaxis

The cost of current anti-D prophylaxis was estimated based on available anti-D IgG products, their UK market share, and the proportion of women receiving prophylaxis. The cost of administering anti-D was based on published estimates [31].

### Financial Benefits of Implementation Scenarios 1 and 2

The incremental cost of moving from the status quo (universal anti-D prophylaxis) to NIPD targeted prophylaxis was estimated. In both scenarios it was assumed that 80% of all RhD negative women will be tested and, as a result, 62% will receive antenatal anti-D; the latter figure allows for the false positive rate (2%) for RhD NIPD tests [16]. A threshold analysis to identify the circumstances under which NIPD might be considered economically attractive compared to RAADP was undertaken for each scenario. For in-house tests, two key cost drivers were identified (royalty fee and price of anti-D IgG). Royalty fees, which are unknown, were varied between zero and an upper figure estimated from commercial kit prices; anti-D IgG costs were varied based on historical price variations and expert opinion about future costs.

### Clinical Consequences and Cost-effectiveness of Implementation Scenarios 1 and 2

The consequences of implementing scenarios 1 and 2 were estimated. These included consideration of parameters such as: false negative and false positive rates; additional sensitisations; and risk of infection from anti-D blood product. Since definitive prospective trial outcomes data is not available for all parameters, some drew on international experts and a review of the literature [13,15]. The incremental cost per change in effectiveness (increased number of sensitisations) was estimated for different test sensitivities.

## Results

### Test costs

Table 1 provides a breakdown of NIPD unit testing cost for a national programme using in-house tests. This basic cost of £16.25 per patient sample represents a *minimum *because it excludes any royalty fee.

**Table 1:** Base Case: Unit Test Cost^1 ^(In-House Laboratory Protocol)

Item	Cost per sample (£)
Blood sampling (phlebotomy)	3.00 ^**2**^

Sample transport & registration	1.10 ^**3**^

Laboratory consumables	4.22^**4**^

Laboratory equipment	1.02 ^**5**^

Laboratory labour	2.07

Result reporting	1.00

Re-tests	0.29

Laboratory overheads	3.55

**Unit Test Cost**	**16.25**

^**1 **^Figures excluding any royalty fee.

^**2 **^See [29]

^**3 **^Based on current charges in West Midlands for mailing 4 samples. Figure will be lower if existing transportation system used; and higher if < 4 samples mailed.

^**4 **^Based on 88 samples per run, includes 20% discount for bulk purchase.

^**5 **^Assumes 44,000 samples processed annually per set of equipment

Use of a commercial kit is calculated to increase testing costs to £46.50 per patient sample. If, as indicated with this kit, a second confirmatory test is required later in pregnancy for all RhD negative results, this would increase costs further to an average of more than £65 per patient. Other test costs were estimated at: postnatal serology test (£3.78); Kleihauer test (£2.87); and associated phlebotomy cost (£3).

### Cost of Status Quo Antenatal Anti-D Prophylaxis

Table 2 presents a breakdown of the annual cost of RAADP in England and Wales (£3.37 million). This is based on the market share for different manufacturers' products, current prophylaxis protocol (500iu BPL; 1250iu Baxter), and a cost of administering prophylaxis at £5 per dose [31]. If a single-dose policy (1500iu) were to become widespread (NICE 2008), this would reduce annual RAADP costs to £2.88 million assuming similar uptake levels.

**Table 2:** Annual Cost for Routine Antenatal Anti-D Prophylaxis (England & Wales)

Product	Market Share (%)	**1**^**st **^**Dose (Injections/Year)**	**2**^**nd **^**Dose (Injections/Year)**	Cost per Dose (£)	**Total Cost for RAADP **^**1 **^**(£/year)**
D-Gam (BPL) 2 × 500 IU	48	35,896	34,731	19.50 ^**2**^	1,730,362

D-Gam (BPL) 1 × 1500 IU	1	748	N/A	33.50	28,791

Rhophylac (CSL) 1 × 1500 IU	40	29,913	N/A	33.50	1,151,654

Partobulin (Baxter) 2 × 1250 IU	11	8,226	7,959	23.35	457,236

**Total**		74,783	42,691		**3,368,044**

^**1 **^Based on an estimated 74,783 women receiving routine antenatal prophylaxis.

^**2 **^Bulk of BPL product used in NHS hospitals purchased at price of £ 19.50/500iu [Dr E Gascoigne, Bio Products Laboratory (BPL), UK, personal communication]. BPL list price of £27/500iu dose is only applicable to private hospitals.

### Financial Benefits of Implementation Scenarios 1 and 2

**Figure 1:**
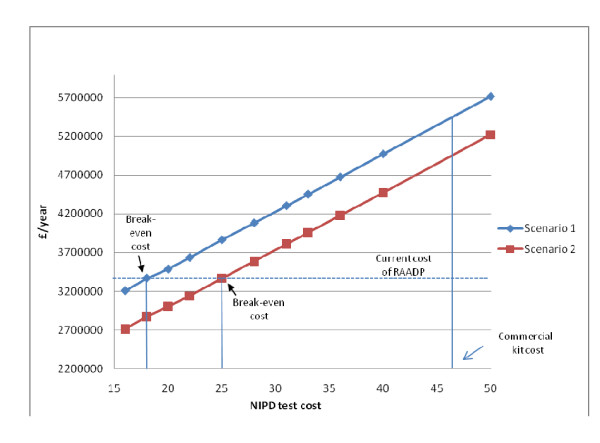
**Annual Cost of Scenarios 1 and 2 for Different NIPD Unit Test Costs vs. Cost of Current RAADP Policy**.

Figure 1 presents the annual cost of Scenarios 1 and 2 at varying unit test costs compared to the annual cost of RAADP (£3.37 million). For in-house tests, threshold analysis shows that Scenario 1 will be less expensive than current RAADP (cost saving) as long as the total NIPD testing cost is below £18.43 per woman. Scenario 1 is predicted to produce a national saving of 5% (£162,258) for in-house tests with a zero royalty fee (£16.25); and virtually no cost saving if the royalty fee is £2.00 (threshold fee is £2.18 per sample). If a single-dose policy were to be introduced, the threshold cost would fall to £15.95 per sample, indicating that NIPD will not be cost saving *even *at a zero royalty fee. In Scenario 2, there are potentially greater savings because NIPD now displaces post-natal testing as well as reducing antenatal prophylaxis. In this case, at a £2.00 royalty fee the annual saving predicted for the current two-dose policy rises to 15% of annual RAADP costs (£507,154) with a threshold royalty fee of £8.83. Once again, if a single-dose policy were to be universally accepted annual savings would fall to £321,993 driving the threshold royalty fee to £6.35. Although laboratory throughput was found to have minimal impact on scenario costs, changes in anti-D IgG prices could influence whether scenarios generate cost savings. For Scenario 1, at zero royalty fee, as long as the average price of anti-D IgG per case is above £28.67, implementation of NIPD remains financially favourable. For Scenario 2, the comparable figure is £39.70. Anti-D IgG prices have remained relatively stable over the last 6 years and are not expected to show significant changes in the mid-term [Dr E Gascoigne, Bio Products Laboratory, UK, personal communication].

Figure 1 shows that the unit test cost of the commercial kit (£46.50) cuts the line for both scenarios significantly above the cost of RAADP and therefore is not financially attractive, even if a repeat test were not required later in pregnancy.

### Clinical Consequences and Cost-effectiveness of Implementation Scenarios 1 and 2

Although annual cost savings of up to £162,258 (Scenario 1) and £656,720 (Scenario 2) are potentially feasible with zero royalty fee, any financial benefit should be considered against possible clinical consequences following NIPD introduction. For example, if an RhD positive fetus is misdiagnosed as RhD negative, due to sub-optimal RhD NIPD test performance [8,9,11-14], there will be an increased risk of sensitisation (0.9-1.5%) due to antenatal prophylaxis being withheld in these cases [32,33]. Table 3 shows the number of additional sensitisations predicted in England and Wales at different NIPD test sensitivity levels for a two-dose policy. In Scenario 1, women who miss their antenatal prophylaxis will still receive postnatal prophylaxis so there will be no further added risk of sensitisation. However, in Scenario 2 (where postnatal serology tests are eliminated) these same women will now not receive anti-D post-delivery. The risk of sensitisation in such cases rises significantly to 13.2% [3]. Table 3 shows that, in Scenario 2, if sensitivity falls below 95% an additional 744 women could become sensitised in England and Wales each year.

**Table 3:** Additional Sensitisations (England & Wales) for Different Test Sensitivities

	**Additional Sensitisations/Year **^**1**^		**Incremental Cost-Effectiveness Ratio **^**2**^	
**RhD NIPD sensitivity **^**3**^	**Scenario 1 **^**4**^	**Scenario 2 **^**5**^	**Scenario 1 (£/sensitisation)**	**Scenario 2 (£/sensitisation)**

94.8% ^**6**^	54	744	3,005 (235)	883 (682)

96.0%	42	573	3,863 (302)	1,146 (885)

97.0%	31	430	5,234 (409)	1,527 (1,179)

98.0%	21	283	7,727 (604)	2,320 (1,793)

98.4%	17	226	9,545 (747)	2,906 (2,244)

99.0%	10	141	16,226 (1,269)	4,658 (3,597)

99.2%	8	113	20,282 (1,586)	5,812 (4,488)

99.4%	6	85	27,043 (2,115)	7,726 (5,966)

99.7%	3	42	54,086 (4,231)	15,636 (12,075)

99.9%	1	14	162,258 (12,692)	46,908 (36,225)

^1 ^Assuming current serology is 100% accurate.

^**2 **^Incremental cost i.e. £ sterling saved per additional sensitisation produced. Figures in brackets ( ), assumes royalty fee negotiated at £2.00 per test.

^3 ^Sensitivity range based on values reported in literature [13,16]

^4 ^Antenatal anti-D prophylaxis directed by RhD NIPD results & postnatal anti-D by serology (1.3% risk) [2]

^5 ^Postnatal anti-D prophylaxis based on RhD NIPD antenatal result (13.2% risk) [3]

^**6 **^Meta-analysis figure for diagnostic accuracy [13]

The figures for different test sensitivities are compared with the predicted incremental cost saving in the final columns, both with a zero royalty fee and a fee of £2.00 per sample. If mass testing is introduced as described in Scenario 1 and a test sensitivity of 99.7% can be achieved [16], this will mean that £54,086 can be saved at the price of each additional sensitisation, as long as a *zero royalty fee *applies. If a fee of £2.00 per sample is incurred, similar to that negotiated for *Q-PCR *by the NHS at £2.70 per test single marker [17] then this figure falls to £4,231 saved per additional sensitisation. For Scenario 2, similar figures are a saving of £15,636 or £12,075 per additional sensitisation respectively. If we include the extra cost incurred for management of sensitisations, most recently estimated at an average £2,885 per person [19], Scenario 2 will generate one additional sensitisation for every £9,190 saved at this royalty fee.

## Discussion

Management of non-sensitised RhD negative pregnancies has become considerably more effective over time with the sequential introduction of post-delivery and antenatal anti-D prophylaxis [6]. Residual RhD sensitisations still occur, mainly due to poor prophylaxis implementation, administration errors and sub-optimal patient compliance [5,19,34]. Advocates of mass RhD NIPD testing therefore focus on the potential for generating net cost savings although this is not quantified [16,18].

The cost analysis presented in this paper shows that the net financial benefit of implementing mass NIPD testing as an add-on using in-house tests (while maintaining current postnatal testing) will be negligible in England and Wales. The level of royalty fee negotiated will be a major influence on this with zero saving predicted above a fee of £2.18 per test. If antenatal NIPD were to also displace postnatal testing, higher net savings could be realised and the royalty fee might rise to nearly £9 per test before these disappear. Use of a commercial kit, such as that currently being marketed in Europe [30] would make NIPD more expensive in both implementation scenarios. Furthermore, if single-dose prophylaxis, now recommended as equally effective by NICE [6], were to become widespread only the second scenario (displacing postnatal testing) would produce a net saving.

Our analysis also indicates that NIPD implementation is unlikely to produce important clinical benefits. The number of sensitisations will not fall appreciably and in fact might rise if NIPD test sensitivity is below 99.9%. This is especially true for the scenario in which postnatal blood cord serology is eliminated in order to maximise any net cost saving. Introduction of NIPD typing must also meet the needs of testing in a diverse population [35]. In the UK, the Race Relations Amendment Act 2000 lays a statutory duty upon the NHS to consider the implications of any new policy on racial equality [36]. In white Caucasians, the most frequent RhD negative genotype is caused by an *RHD *deletion, while in Black Africans and South Asians other variants exist which can result in mistyping [37]. There has been a recent call for careful consideration of patients' ethnic background by obstetricians [38]. Antenatal prophylaxis has been reported to be equally effective, and also more cost-effective, in ethnic minority populations in the UK and elsewhere [19,23,39,40]. However, to date NIPD assays have primarily been validated in white Caucasian populations and their reliability in ethnic minority populations still has to be demonstrated [41]. Due to the absence of a 'gold standard' comparator, it is not possible to establish the absolute accuracy of the serology test, although since the test is performed manually it will be prone to some human error. Thus, even in scenario 1, sensitisations may occur because of inaccuracy in the postnatal serology test. Two recent studies have suggested that RhD NIPD may provide more accurate results than postnatal serology, both by minimising human error and by helping to detect D variants and weak D newborns [14,28].

Based on our findings it is difficult to argue that NIPD targeted antenatal prophylaxis will be sufficiently cost-effective to warrant its large scale introduction. Increased magnitude of savings will be at the expense of additional sensitisations. Even the maximum amount of £9,190 saved per additional sensitisation is much lower than the figure of £15,903 - £17,668 judged acceptable for avoiding a sensitisation by NICE [42].

However, if NIPD testing could be carried out earlier in pregnancy cost-effectiveness might be improved. For example, adherence to prophylaxis guidelines for 'high risk' antenatal events might be improved if women are identified as carrying an RhD positive child early in their pregnancy [43,44]. Balanced against this is the fact that NIPD test accuracy is known to deteriorate at lower gestational age [45-47]. The accuracy of mass testing in early pregnancy is uncertain. A large scale UK trial of NIPD testing in the first trimester of pregnancy has still to report [Dr. Kirstin Finning, National Blood Service, Bristol, UK, personal communication]. However, a US company which has recently launched a fetal *RHD *genotyping test for use in first-trimester pregnancies reports a sensitivity of 97.2% [48].

During the development of the original 2002 NICE guidelines, questions were raised about the safety of anti-D IgG because of isolated cases of hepatitis C infection in the early 1990's [49,50]. Avoidance of unnecessary use of anti-D has therefore been suggested as a 'soft benefit' for NIPD even though anti-D continues to be judged safe for routine antenatal use by NICE [1,6]. Modern processes make it among the lowest-risk biological products in use [51], especially in combination with avoidance of high risk plasma donors [52-54]. A second linked argument is that NIPD might improve prophylaxis coverage [4]. Some authors have suggested that low uptake might be linked to anxiety about anti-D blood products [4,5,55]. At the same time, it is recognised that poor antenatal attendance, knowledge that the father is RhD negative, or certainty that this is a woman's last pregnancy influence uptake [19]. Such factors would be unaffected by introduction of NIPD.

A final advantage put forward is linked to anti-D supply. In England and Wales, where anti-D IgG is produced using plasma purchased from the USA, there have been no supply problems. However, in countries such as the Netherlands where in-country sourcing requires the purposeful hyperimmunisation of volunteers, decreased use of anti-D has been viewed as an ethical benefit in its own right [56]. Scientists are currently working on the development of recombinant anti-D IgG as a replacement for human plasma products [57]. Although there is no completely satisfactory product on the market [58], a phase 2 clinical trial has recently commenced [59]. In future, this has the potential to address these concerns [60,61]. If, as is likely, the final recombinant product is more expensive than the human product then NIPD would become financially more attractive by generating greater anti-D savings.

The main limitation in the present study is uncertainty over the level of royalty fee for in-house NIPD tests. The speed of development and diffusion of recombinant anti-D IgG may also affect the conclusion drawn, but this is difficult to estimate without information on the eventual cost of this product. Further new technologies which might influence cost-effectiveness include NIPD markers for conditions such as haemoglobinopathies or Trisomy 21 (Down's syndrome) which appear to be nearing final development [62-66]. If a battery of markers, including fetal *RHD *status, were combined in a single NIPD test then this would reduce costs through economies of scale in common processes such as DNA extraction and amplification. Finally, our economic analysis, which only considered England and Wales, may be less applicable in other countries. For example, in some European countries there is no policy of routine antenatal prophylaxis although this is now being considered [67,68]. In such cases, the introduction of targeted prophylaxis underpinned by RhD NIPD testing might offer similar clinical benefit to introduction of RAADP at a potentially acceptable cost.

## Conclusion

Our analysis does not support routine implementation of RhD NIPD testing in England and Wales at present for non-sensitised pregnancies. Annual savings will be relatively small and balanced against this increased maternal sensitisations may be unacceptably high. First trimester testing is unlikely to alter this picture significantly although other emerging technologies may. There is also a need to demonstrate the reliability of assays in different ethnic minority populations.

## Competing interests

The authors declare that they have no competing interests.

## Authors' contributions

AS, LO and KF conceived the idea for the study. LO obtained the data and completed the analysis. AS and LO wrote the manuscript which was reviewed by all authors. All authors had full access to all data in the study and can take responsibility for the integrity of the data and the accuracy of the analysis. All authors read and approved the final manuscript.

## Pre-publication history

The pre-publication history for this paper can be accessed here:

http://www.biomedcentral.com/1471-2393/11/5/prepub
